# PB.30: What is your biopsy threshold in symptomatic clinic and what should it be? B2:B5 biopsy ratio: 18 months in our centre

**DOI:** 10.1186/bcr3530

**Published:** 2013-11-08

**Authors:** R Gray, G Porter, J Steel

**Affiliations:** 1 Plymouth Hospitals NHS Trust, Plymouth, UK

## Introduction

While a <2% benign biopsy rate is widely regarded as the gold standard in the screening population, there is no such guidance for biopsy in symptomatic patients. When symptomatic patients present with a lump visible on ultrasound, the decision to biopsy is down to the individual radiologist. There is currently no gold standard for the benign:malignant biopsy ratio.

## Methods

A prospective list of all symptomatic clinic biopsies was compiled over 18 months from January 2012 to May 2013. Imager and histological outcome of each biopsy (B1 to B5) were recorded. Cancer detection rate for each operator per 1,000 symptomatic cases was also calculated. Axillary biopsies were excluded.

## Results

Six anonymised operator biopsy results were recorded. There is a wide variation with our centre of B2:B5 ratios without significant variation in the cancer detection rate between operators.

## Conclusion

This study demonstrates considerable variation in biopsy threshold within one unit. There is no correlation of biopsy rate and cancer detection in this dataset, meaning that potentially there are many unnecessary biopsies performed. This study has stimulated discussion in our unit, and is a useful and simple audit to perform to investigate biopsy threshold. If performed by a number of units nationally, it may be possible to assess the range for these parameters and to suggest a normal range.

